# Empathic Skills Training As a Means of Reducing Cyberbullying among Adolescents: An Empirical Evaluation

**DOI:** 10.3390/ijerph20031846

**Published:** 2023-01-19

**Authors:** Ashraf Atta M. S. Salem, Amthal H. Al-Huwailah, Mahfouz Abdelsattar, Nadiah A. H. Al-Hamdan, Esraa Derar, Sheikhah Alazmi, Mosaad Abu Al-Diyar, Mark D. Griffiths

**Affiliations:** 1 College of Management Sciences, Sadat Academy for Management Sciences, Alexandria 21578, Egypt; 2 College of Social Sciences, Kuwait University, Kuwait P.O. Box 68168, Kuwait; 3 Hurghada Faculty of Education, South Valley University, Hurghada 84511, Egypt; 4 Evaluation and Testing Unit, Ministry of Eduction, Qurain P.O. Box 47041, Kuwait; 5 Department of Psychology, College of Arts, Suez University, Suez 43533, Egypt; 6 International Gaming Research Unit, Department of Psychology, Nottingham Trent University, Nottingham NG1 4FQ, UK

**Keywords:** empathic skills training, cyberbullying, cognitive-behavioral counselling, adolescents, empirical evaluation

## Abstract

Cyberbullying is a form of aggression in which electronic communication such as e-mails, mobile phone calls, text messages, instant messenger contacts, photos, social networking sites and personal webpages are used to threaten or intimidate individuals. Cognitive-behavioral therapy (CBT) counselling based on empathic training may reduce cyberbullying among adolescents. The present study investigated the impact of developing empathy skills in reducing cyberbullying among a sample of adolescents using two groups (i.e., an experimental group and control group). The experimental group received counselling intervention based on CBT with special focus on improving empathy whereas the control group received CBT general counselling. The participants comprised 217 adolescents (experimental group = 98 adolescents, control group = 119 adolescents) with a mean age of 15.1 years (SD ± 1.5). The measures included the Toronto Empathy Questionnaire (TEQ) and the Bullying, Cyberbullying Scale for Adolescents (BCS-A). Results showed that there were statistically significant differences on TEQ scores and BCS-A scores in the experimental and control groups after the intervention but more so in favor of the experimental group in terms of reduced levels of cyberbullying (both victimization and perpetration). Positive gains among the experimental group in both empathy and reduced cyberbullying remained at two-month follow-up. It is recommended that teachers and school counselors tackling cyberbullying should use empathy training as part of their cyberbullying prevention programs.

## 1. Introduction

Adolescence is a crucial developmental period during which an individual undergoes social, emotional and physical changes that can lead to poor self-perceptions [1]. The world’s adolescent population is estimated to be 1.2 billion [2,3]. Adolescents may experience problems during this time that affect both their personal and social growth [4]. Social problems that adolescents confront can be the result of negative parental control and peer pressures and in some cases can lead to aggressive behavior [5,6].

Emotions play an unparalleled role in teenage behavior [7,8] and behavioral problems among adolescents are risk factors in the development of internalizing issues such as anxiety and depression [9]. Furthermore, the co-occurrence of behavioral, emotional and cognitive issues can cause increasing worry [10]. One such behavioral problem is aggressive behavior. There could be underlying causes for this aggression, such as adaptive or maladaptive emotional states [11,12]. Aggressiveness has emotional and motivational elements. When seen emotionally, aggression is often the result of imminent fury [13].

Advances in communication technologies have opened up new avenues in the form of cyberbullying among adolescents [14,15,16]. By joining groups, publishing images and videos and commenting on others’ posted content, social networking sites can unintentionally enable and maintain cyberbullying [17]. Cyberbullying is conceptually different from typical school bullying [18,19,20,21]. Cyberbullying may be induced by the lack of respect for social values, the absence of parental follow-up, poor guidance of parents and teachers and/or by heavy pressure put on the bullies themselves [22,23].

### 1.1. Cyberbullying: Second Generation of Bullying

Cyberbullying is an extension of traditional bullying and occurs when electronic communication is used to threaten or intimidate individuals [24,25]. Cyberbullying has distinct qualities that are different from traditional bullying, such as the possible anonymity of bullies, the accessibility of victims, and a potentially wider pool of victims. Cyberbullies are frequently unknown or virtually anonymous and they can reach their victims 24 h a day, seven days a week, wherever the target may be, and their audience may be anywhere worldwide [14,26,27,28,29,30,31,32].

Cyberbullies intimidate, humiliate and taunt victims [33,34,35,36,37]. Cyberbullying is a phenomenon which can be manifested in the following forms/types: (1) “catfishing” or luring people into long-term emotional or romantic relationships through fake online identities and social networks; (2) cheating in MMOGs, establishing roving gangs and blocking entrances; (3) sending insulting, humiliating, or threatening messages or photos to the harassed party; (4) “flaming”, comprising online, aggressive and hostile internet impersonation; (5) internet “slamming” or “bystander” harassment; (6) using remote administration tool software to spy on and access the targeted person’s computer or camera without their consent; (7) relational aggression (spreading rumors, creating a false *Facebook* page, removing the victim from a friend list, or posting cruel remarks or threats on the victim’s *Facebook* wall); (8) sexting (sending embarrassing, sexually explicit images); (9) shock trolling (offensive internet messages or statements designed to anger, frustrate, or disturb); and (10) stalking people online and threatening violence, making the victim scared and/or worried for their safety [38,39,40,41].

Despite being linked to many negative consequences, few studies have examined cyberbullying risk factors and predictors, especially among adolescents. Personal views may predict cyberbullying and other online behavior [42,43]. While there has been relatively little research on school-based cyberbullying, some preliminary data suggest that personality traits such as self-esteem [44,45], locus of control [46], self-efficacy [47] and neuroticism [48] may affect behavior such as aggression.

Cyberbullying has adverse consequences on both cyberbullies and their victims. Hinduja and Patchin [49] argue that cyberbullying causes emotional suffering. Effects vary by person and kind of cyberbullying [50]. Moreover, cyberbullying can cause low positive and high negative affect [51]. Cyberbullying is typically an online version of traditional bullying [51,52]. One study [53] reported that cyberbullying makes teenagers angry, ashamed and indifferent. Cyber-abuse victims were reported to be enraged (56%), wounded (33%), ashamed (32%) and terrified (13%). Females, especially 13- to 15-year-olds, reported these feelings more often.

What exaggerates the severity of cyberbullying is that victims are unable to remove offensive content once it has been posted online by the perpetrators. Bullying practices in the virtual world frequently reach a wider audience because they are often performed in the presence of wide range of people online [54,55,56]. As a serious behavioral problem, cyberbullying affects the lives of adolescents in a profoundly negative way [55].

Cyber-victimization has negative effects on physical, social and cognitive functioning, development and well-being, as well as psychological, academic and emotional issues such as depression, suicidal ideation, truancy, school problems and deviant behaviors [37,57,58]. Even though cyberbullying is an important research issue, little is known about the dynamics of perpetration and victims [59]. According to several empirical studies, cyberbullying can have substantial physical and psychological consequences, such as psychosomatic and depressive symptoms, anxiety, self-harming behavior and substance misuse [60,61,62,63]. Consequently, to deal with cyberbullying and its implications, preventative and intervention programs are required [64,65]. To build these programs, specialized study is required to understand the individual and social dynamics that influence cyberbullying participation.

According to Modecki et al. [66], mean prevalence rates are 15.2% for cyberbullying victimization and 15.5% for cyberbullying perpetration based on meta-analytic data. In Egypt (where the present study was carried out), the majority of studies on school violence have focused on children from urban communities [67,68]. With regard to the prevalence of cyberbullying in Egyptian society, a study by Arafa and Senosy [69] reported that almost half of students reported experiencing cyberbullying victimization in the past six months (48.2%). Female students, students living in urban areas and those who spent more hours using the internet reported significantly more exposure. Harassment was the main type stated by females (79.8%), while flaming was most reported by males (51.8%). Students felt anger (63.1%), hatred (23.2%) and sorrow (22.6%) regarding the worst victimization incidents.

In addition, literature reviewed relating to the prevalence of cyberbullying in Egypt is incomplete and to some extent gender-biased. The study by Hassan et al. [70] reported that 41.6% of female adults were exposed to cyber-violence in the past year and 45.3% of them were exposed multiple times. The most common method of exposure was via social media and the offenders were unknown to 92.6% of victims. Additionally, it was reported that 41.2% reported receiving images or symbols with sexual content, 26.4% received insulting e-mails or messages, 25.7% received offensive or humiliating posts or comments, 21.6% received indecent or violent images that were demeaning to women, and 20.3% received infected files via e-mail. The majority of victims (76.9%) experienced psychological effects such as anger, anxiety and fear, 13.6% social effects, 4.1% physical harm, and 2.0% financial losses. Blocking the offender was the most common response among victims. The study concluded that females in the Egyptian population are highly exposed to cyber-violence. Therefore, it is crucial to implement an anti-cyber violence program to combat this phenomenon.

These rates are lower than those of Arafa and Senosy [71], who reported that 52.9% of female participants in their study experienced cyberbullying, with 69.9% reporting more than a single incident. Additionally, they reported that cyber-harassment was the most prevalent type. Another study by Arafa, et al., [69] stated that almost 80% of all female students surveyed reported experiencing cyber-sexual harassment in the previous six months, with the majority of victims experiencing it multiple times. Students who lived in cities and students who used the internet for more hours per day reported higher levels of exposure. The harassed students reacted to the harassment incidents primarily with anger (65%), fear (20.1%), hatred (18.5%) and sorrow (18.4%). The literature reviewed lacks comprehensiveness, as to some extent it is gender-biased because it tends to focus on specific categories in society, such as females or nurses. Therefore, the present study focuses mainly on cyberbullying among adolescents and the usefulness of an empathy-based intervention program to reduce the problem.

### 1.2. Cognitive Behavior Therapy (CBT): Effective Intervention Approach

Cognitive behavior therapy (CBT), which incorporates both behavioral and cognitive techniques to support behavior change, is one therapeutic approach that is currently attracting interest due to its reported empirical testability, efficacy, and cost-effectiveness [72,73,74]. In this context, using a CBT framework rather than other therapeutic models is encouraged because CBT can be used to inform brief interventions for individuals or larger groups. Beck’s [75] cognitive-behavioral theory of emotion inspired CBT. According to this theory, emotions arise from how events are appraised or interpreted, which is influenced by underlying cognitive structures that cause faulty or biased interpretations of events.

### 1.3. Empathic Skill for Reducing Cyberbullying

Empathy is a fundamental human personality trait that is thought to facilitate social interactions and interpersonal communication in at least two ways. First, affective empathy is natural and allows individuals to assimilate and display compassion in response to other people’s emotional states. Second, cognitive empathy requires more conscious deliberation and allows individuals to understand and display compassion in response to other people’s emotional states. This distinction is supported by both self-reported measures and neuroscientific evidence [76,77,78]. Empathy is individuals’ awareness of passions and positive emotions in themselves and others, and affects the social interaction based upon that [79].

Several studies have shown that CBT improved empathic skills in various populations. A study by Song et al. [80] reported that CBT improved empathy among patients with chronic pain, independently of its effect on pain, suggesting that CBT is useful for improving interpersonal relationships in this group. Cognitive behavior therapists investigate clients’ thoughts, feelings, and behaviors in a variety of situations (including reactions to the therapist) along with relevant childhood experiences to understand underlying core beliefs and conditional assumptions. Empathy assists them in understanding both emotional reactions and the meanings of experiences, as well as how these elements are interconnected in a specific client. Effective listening requires an understanding of transference and countertransference. Empathy may aid in the recognition and understanding of transference and countertransference, as well as their appropriate application during therapy. Empathic ability may be part of sensitivity to an individual’s own feelings, including countertransference feelings, which should prevent countertransference acting out [81,82,83,84].

Low empathy has been associated with a high level of participation in antisocial behaviors such as bullying, cyberbullying, vandalism, stealing, assault, substance abuse, and status crimes. Therefore, promoting empathy in schools appears to be beneficial in combating these behaviors [85]. It has been also shown that lack of empathy and poor friendship quality have a detrimental impact on cyberbullying. Consequently, empathy and friendship quality are characteristics that prevent adolescents from developing cyberbullying behavior [86]. In addition, it has been shown that there is a long-term association between lack of empathy, social–emotional problems, and cyberbullying. Moreover, in one study, neither cyberbullying nor cyber-victimization predicted social withdrawal or psychopathological symptoms [87].

A review of previous cyberbullying intervention research noted specific flaws in these studies, highlighting the importance of CBT-based interventions for reducing cyberbullying. A study by Barkoukis et al. [88] examined the impact of a cyberbullying intervention program that targeted the psychosocial risk factors for cyberbullying during adolescence. However, the outcome measure referred to intent to cyberbully others rather than actual cyberbullying behaviors. In addition, Foshee et al. [89] investigated the influence of the program *Moms and Teens for Safe Dates* on several victimization outcomes, including cyber-dating-abuse, but this is not directly comparable to cyberbullying behaviors. Therefore, the present study fills a research gap because it focuses primarily on preventing cyberbullying by fostering empathy among a sample of adolescents.

Due to a lack of empathy, Ramdhani [90] found that there is an online disinhibition effect, which can lead to adolescent cyberbullying. Empathy works as a social anchor, preventing antipathic behavior among adolescents during face-to-face interactions. Empathy is thought to be effective in reducing adolescents’ involvement in cyberbullying experiences especially as perpetrators [91,92]. According to Krumbholz and Scheithauer [93], cyberbullying perpetrators and victims have lower empathy levels than those who do not engage in cyberbullying. Steffgen et al. [94] found the same thing, with adolescent cyberbullying perpetrators scoring much lower on empathy than those who had never been involved in cyberbullying. Evidence has steadily accumulated regarding the negative consequences of student involvement in cyberbullying to the extent that it may lead victims to commit suicide [95]. Consequently, more research is required to improve the understanding of the long-term effects of cyberbullying on adolescent health [59].

### 1.4. The Significance of the Present Study

Cyberbullying is a major concern that has a severe influence on adolescents’ mental health and academic performance, which is why this research is so important. Because adolescence is such an important developmental stage, the present study’s participants were selected from this population. Furthermore, the research contributes significantly to the literature of cognitive sciences in the Arab region. There is a scarcity of studies on this topic, particularly in the Egyptian context.

### 1.5. The Purpose of the Present Study

The main objective of the present study was to assess the impact of using empathic skills training in alleviating cyberbullying among a sample of adolescents from Egypt. It is an empirical evaluation of the effectiveness of cognitive-behavioral counselling based on supporting empathy in reducing the adverse consequences of cyberbullying among Arab adolescents.

### 1.6. Hypotheses of the Study

The present study evaluated a cognitive-behavioral program based on enhancing empathy to alleviate the symptoms of cyberbullying among adolescents and proposed the following research hypotheses:

**H_1_.**  *The experimental group will have significantly increased empathy scores compared to the control group post-test.*

**H_2_.**  *The experimental group will have significantly reduced cyberbullying (perpetration–victimization) scores compared to the control group post-test.*

**H_3_.**  *Any positive gains made by the experimental group in relation to both increased empathy and reduced cyberbullying will be maintained at two-month follow-up.*

## 2. Materials and Methods

### 2.1. Methodological Note

Cognitive-behavioral therapy (CBT) counselling based on empathic training may reduce cyberbullying in adolescents. The present study investigated the impact of developing empathy skills to reduce cyberbullying among a sample of adolescents using two groups (i.e., an experimental group and control group). The experimental group received counselling intervention based on CBT with special focus on improving empathy whereas the control group received general CBT counselling.

### 2.2. Participants

The study sample comprised 217 adolescents (Grades 7 and 8) aged between 12 and 16 years (M_age_ = 15.1 years; SD ± 1.5 years) in intermediate schools in Alexandria, Egypt, from predominantly middle-class neighborhoods. Adolescents self-identified as males (49%) or females (51%). The sample was divided into two groups, the experimental group comprising 98 students and the control group comprising 119 students. Participants were intentionally chosen after they had completed psychometric scales assessing empathy and cyberbullying. The main inclusion criterion was that they should have the lowest scores on the empathy scale and the highest scores on the cyberbullying scale. The two groups were matched in terms of gender, age, intelligence, empathy and cyberbullying (see Table 1).

### 2.3. Measures

*Bullying and Cyberbullying Scale for Adolescents* (BCS-A): The BCS-A [96,97] comprises 26 items and two subscales (Victimization Scale [13 items] and a Perpetration Scale [13 items]). Items (e.g., “*Punched, hit, kicked, pushed, or shoved me, on purpose*” (offline victimization) and “*Sent or posted, mean or hurtful pictures/videos about me*” (online victimization]) are scored from 0 (*never*) to 4 (*always*) and the scoring range is 0 to 104. High scores indicate higher levels of cyberbullying [96]. Cronbach alphas in the present study were 0.87 for victimization, 0.93 for perpetration and 0.89 for the whole scale.

*Toronto Empathy Questionnaire* (TEQ): The TEQ [98,99] was used to assess empathy. It comprises 16 items (e.g., “*When someone else is feeling excited, I tend to get excited too*”) and they are scored on a five-point scale ranging from 0 (*never*) to 4 (*always*). Items 2, 4, 7, 10, 11, 12, 14 and 15 (e.g., “*Other people’s misfortunes do not disturb me a great deal*”) are negatively worded and are reverse scored. All responses are summed to generate a total score out of 64. Higher scores indicate greater empathy. Cronbach alpha in the present study was 0.94 for the whole scale.

### 2.4. Procedure

The study adopted a quasi-experimental research method based on assessing the expected changes concerning the dependent variables (i.e., empathy and cyberbullying) based on the techniques of the cognitive-behavioral intervention on the independent variable (empathy). Approval for the study was granted by the second author’s university ethics committee (Suez Canal University). Recruitment began by sending an email to a list of ten middle schools in Alexandria. For the schools that agreed to participate, the principal investigator met with the school principal and teachers to introduce them to the study, explain how adolescents could participate, the time commitment for the study, and what adolescents would be expected to do if they were to participate.

After the meeting, classroom announcements were made to participants to inform adolescents about the schedule of the study, what they would be expected to do if they were to participate, and their rights as participants, including explanation of the confidentiality of their results and the ability to withdraw from the study at any time without penalty. There were 260 parental permission slips passed out to the Grade 7 and 8 students. Of these, 217 parents/guardians agreed to allow their children to participate, four declined participation, and the remaining six were never returned. Data collection occurred over two days.

The official visits to the target school were to obtain the full data for the participants and to get them to log into the study activities. After obtaining the official data from the school, participants completed the e-version of the questionnaires on cyberbullying perpetration, cyberbullying victimization, and empathy. The actual intervention program was delivered via an online meeting program (*Zoom* online meetings) to cope with the precautionary procedures and social distancing posed by the government due to the COVID-19 pandemic. After intervention, participants post-tested using the e-version of the scales to compare the mean scores of the pretesting and the post-testing of the study tools in order to determine the actual impact of the intervention. Assent was obtained by adolescents prior to data collection. The present study investigated the impact of developing empathy skills to reduce cyberbullying among two samples of adolescents (i.e., experimental group and control group). The experimental group received counselling intervention based on CBT with special focus on improving empathy, whereas the control group received CBT general counselling.

### 2.5. Cognitive-Behavioral Therapy

The interventions that adolescents received were conducted over several sessions as follows: 20 sessions divided into two sessions per week and each session ranging from 45 to 60 min. Table 2 outlines the counseling methods used in the program:

## 3. Results

Table 3 shows that there were differences in the mean scores of the empathic skills between the pre-testing for the experimental group on the empathy and cyberbullying scales in comparison with post-testing. The *t*-test scores were significant at the *p* < 0.01 level which demonstrates the positive impacts of the empathy-based intervention on reducing the cyberbullying levels among adolescents.

**Figure 1:**
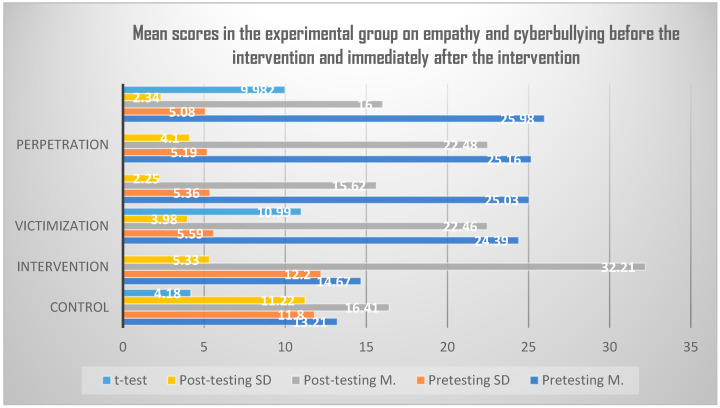
Graphic representation of the first hypothesis.

There were also statistically significant differences between the mean scores of the experimental group members in the pre-and post-measurement on the TEQ (average of the pre-measurement was higher) and on the BCS-A (average of the post-measurement was higher). Table 4 also shows that the positive gains among the experimental group in both empathy and reduced cyberbullying remained at two-month follow-up. In the empathy scale, there are differences in the mean scores of the control and intervention groups (M_control_ = 11.41, SD_control_ = 11.22; M_intervention_ = 32.21, SD_intervention_ = 5.33) resulting in statistically significant difference as shown in *t*-value (4.18) at significance level (*p* = 0.002). With regards to victimization, mean scores are different between both control and intervention groups; (M_control_ = 22.46, SD_control_ = 3.98; M_intervention_ = 15.62, SD_intervention_ = 2.25) resulting in statistically significant difference as shown in *t*-value (10.99) at significance level (*p* = 0.0001). The case was similar with perpetration, in which mean scores are different (M_control_ = 22.48, SD_control_ = 4.10; M_intervention_ = 16.00, SD_intervention_ = 2.34) resulting in statistically significant difference as shown in *t*-value (9.98) at a significance level (*p* = 0.0001) (Figure 1).

Table 5 shows that males scored higher as cyberbullies (M_male_ = 14.84, SD_male_ = 0.95, M_female_ = 13.42, SD_female_ = 1.12). and females scored higher as cyber-victims (M_male_ = 13.80, SD_male_ = 0.694, M_female_ = 15.02, SD_female_ = 0.746). Conversely, female adolescents scored higher than their male counterparts on empathy scales M_male_ = 28.56, SD_male_ = 0.813, M_female_ = 31.23, SD_female_ = 0.750).

## 4. Discussion

The present study investigated the effectiveness of empathic skills training in reducing cyberbullying levels among a sample of adolescents in the intervention group. There were significant differences in the scores of the experimental group in the post-testing of both the TEQ and BCS-A.

This finding provides evidence that the proposed CBT intervention had a direct effect in increasing adolescents’ empathy levels and decreasing their levels of cyberbullying. Improvement in adolescents’ empathy which is manifested in reduced levels of cyberbullying (either victimization or perpetration) is attributed to the intervention only, with no intervening or undesirable variables. It is possible that perpetrators do not fully understand the consequences of their online bullying actions because they do not see their victims’ faces in order to explore their feelings, in relation to the online disinhibition effect [20,100,101].

The majority of victims may not know who their attackers are, and only 40% to 50% of cyberbullying victims are aware of the identity of their attackers. Victims may feel frustrated and helpless due to the perpetrator’s anonymity [102,103,104]. It has been shown that cyberbullying perpetration, cyber-victimization, empathy, adaptive and maladaptive cognitive emotion regulation strategies, and moral disengagement are closely related with each other. Cyberbullying perpetration has been shown to be correlated with blame, affective and cognitive empathy, intention to comfort, and moral disengagement. Cyber-victimization has been shown to have a close relationship with self-blame, rumination, acceptance, planning, and cognitive empathy [60,95].

It is worth noting that cyberbullies lack affective empathy and they also lack empathy in the cognitive domain which includes the inability to consider others’ perspectives [60,105]. Cyber-victims lack the capacity to comprehend and experience the emotions of others [60]. However, it appears that the relationship between cyber-victimization and empathic skills is more complex. Several studies [106,107] demonstrate, for instance, that empathy does not explain cyber-victimization among adolescents. In addition, research [54,108,109] indicates that cyber-victims are empathically sensitive to the affective states of others. Cyber-victimization, according to Shannen et al. [110], was significantly correlated with adolescents’ total empathy and cognitive empathy, but not with affective empathy. Cyberbullying has been found to be significantly related to cybervictimization.

In addition to an inability to understand the emotions of others, including in cyberbullies, the present study showed that cyber-victims lack (and struggle with) emotion regulation [111]. According to the Cyclic Process Model [112], if cyber-victimized adolescents are unable to regulate a wide range of negative emotions, such as heightened levels of anger, depression, and distress, this can be a precursor to their proclivity to become cyberbullies. Previous research suggested that cyberbullying is perpetrated due to maladaptive emotion regulation. In general, the findings on the relationships between affective or cognitive empathy and victimization were mixed and less clear [105,113,114], with effects ranging from low negative to non-significant.

Participants showed increased empathic skills after the remedial intervention through empathic skills training that coincides with the findings of several studies [113,115,116,117,118,119,120]. It is evident that there was a decline in the cyberbullying of participants after the counseling remedial intervention, which also concurs with other studies [63,121,122,123,124,125,126,127,128,129].

In the present study, there were gender variations in cyberbullying, with males scoring higher as cyberbullies and females scoring higher as cyber-victims. Conversely, female adolescents scored higher than their male counterparts on empathy scales. These findings are consistent with those of Abu AlDyiar [130], who found that female adolescents exhibit more empathy than males. Cyberbullying is more common among males than females and males are more likely to direct their cyberbullying behavior towards females as their targets.

Students’ noticeable improvement in terms of their scores on empathic skills in the present study is attributed to the activities, exercises, and reinforcement methods that the remedial intervention counseling program contains. These activities appear to be important in reducing the cyberbullying intensity and reinforcing the aspect of normal behavior among adolescents. Since bullies usually target weaker individuals, they do not accept others’ opinions, nor do they accept any discussion with them and they usually harass them physically and/or psychologically. However, bullying behaviors declined markedly among adolescents due to the remedial counseling intervention in the present study, leading to a strengthening of empathy towards others. These results concur with other studies [113,116,117,119,120].

Cyberbullies used activities in the cognitive-behavioral program to learn several types of positive social behavior and demonstrated the importance of the family role in reinforcing promising behaviors and strengthening positive personality traits [62,120,122,125,126,127,128]. Cyberbullies feel less empathy for their victims. Internalized suffering is experienced by cyber-victims, including depression, anxiety and low self-esteem, insecurity, suicidal thoughts, loneliness, low school success, drug addiction, somatic symptoms, and low self-confidence [131,132]. They frequently have an overprotective family background or adverse family environments [133], or they lack family support [134] and they may face a variety of social difficulties, such as peer rejection or poor peer acceptance (e.g., marginalization) [135].

Empathy-based CBT interventions have been found to reduce both traditional bullying [136] and cyberbullying [137]. Improving empathy represents one possible protective factor against negative online behavior as a cyberbully [138,139]. The study results showed that one of the most promising aspects of enhancing empathy was to develop social skills among cyberbullies to allow them to feel cyber-victims’ suffering. The level of cyberbullying among participants decreased due to eradicating behavioral problems and increasing cognitive processes and social skills. These results consort with results of studies conducted in similar contexts. The results of the study by Abu Aldyiar [130] —which was conducted among Egyptian adolescents—showed that both empathy and self-esteem played a crucial role on reducing cyberbullying. In addition, a study by El-Khouly [140] indicated that comprehensive selective counselling based on empathy enhancement proved to be effective in alleviating cyberbullying among adolescents with special needs.

In the present study, positive impacts of the CBT-based intervention were effective for a long time after post-testing, with adolescents still showing low cyberbullying and enhanced empathic skills. Both cyberbullying and empathic skills in the post-testing and follow-up sessions were closely similar because there were no extra interventions. Therefore, CBT effects appear to extend for a period of time. In the long run, CBT is effective in reducing the possibility of repeated cyberbullying. This concurs with the study by Szász-Janocha et al. [141] which reported long-term effects of a manual-based CBT treatment for adolescents suffering from internet use disorders. The results of their study indicated that even a four-session brief intervention can achieve a medium to large effect over 12 months. Moreover, Lee and Lee [142] showed the effects of a CBT-based intervention program for mental health promotion among university students in the follow-up stage. In addition, Chiang, et al. [143] indicated that cognitive behavioral group therapy (CBGT) is effective in the long-term (one year) for patients with depression. Both groups were assessed before, after, and for three, six and twelve-month follow-ups (all occurring within the first three months of treatment). Six months after the sessions ended and one year later, depression had significantly decreased.

## 5. Limitations

There are a number of limitations to the present study. One is that the study was conducted among Egyptian students in a middle eastern context. This should be taken into consideration if researchers elsewhere try to replicate the study in other geographical contexts, because the findings are not necessarily generalizable. These limitations should be carefully considered when evaluating and reproducing the study in other contexts. It should also be noted that the two scales used (TEQ and BCS-A) depend mainly on self-report and are subject to well-known method biases (e.g., memory recall, social desirability, etc.). However, self-report scales are extremely helpful in determining social anxiety disorders. These scales are practical in that they take little time to administer and score. They can be given repeatedly over time to assess the long-term effects of an intervention. In theory, scales eliminate the need for the clinician to interpret patient responses, thereby reducing an important source of error variance. In addition, results showed that the proposed counselling intervention was effective in alleviating cyberbullying behaviors, although the intervention may not have been long enough to change core cyberbullying behaviors. Actual cyberbullying behaviors may not be changed in a short/immediate time, because individuals do not have sufficient time to increase or decrease their behaviors.

## 6. Conclusions

Despite these limitations, the present study contributes significantly to the literature on empathy training interventions for the reduction of cyberbullying. The findings indicate that the empathy-based training program has significant implications from two angles: promoting peer coexistence in the classroom by reducing cyberbullying and increasing adolescents’ empathy in a school setting. Given the association between empathy training, coping strategies, and well-being [144,145], the empathy education program may be a beneficial intervention for adolescent mental health during this critical developmental stage [146].

These findings also help in the development of school-based programs to prevent cyberbullying while also promoting adolescents’ psychosocial functioning and well-being. Furthermore, the findings help to improve the understanding of the mechanisms underlying the effects of empathy-based interventions on adolescents. Based on these findings, it appears that emotional education intervention and prevention programs are appropriate for adolescents and should be incorporated into school educational plans to increase student self-efficacy and decrease behavioral problems [147].

When considering implementing an intervention program to address emotional and behavioral problems such as cyberbullying, teachers and school counsellors may use similar intervention in similar studies to identify adolescents’ social and emotional strengths/weaknesses, interests and concerns. Future studies should determine whether this intervention program is more or less effective for high-risk students, whether it can create a climate of empathy among all types of students, and make adjustments accordingly without jeopardizing the program’s integrity, in order to increase the likelihood of successful implementation [148]. Based on the experiences here and those of others [145], it is proposed that educators consider engaging in empathy-based training interventions before teaching students about these skills. This would allow for greater understanding of the impact of empathy training development compared to previous studies using short-term interventions and cross-sectional data [105,149]. Furthermore, the present study adds to the existing body of research that supports the role of empathy-based training in reducing the prevalence of cyberbullying behaviors and increasing adolescent life satisfaction [150,151,152].

## Figures and Tables

**Table 1:** Mean scores and values of (U) and (Z) in the variables of the study (age, socioeconomic class, general intelligence, empathy and cyberbullying) for the control (*n* = 119) and experimental (*n* = 98) groups prior to the intervention.

Variable			Control		Experimental		Mann-Whitney U	Z	Asymp. Sig. (2-Tailed)
			M.	SD	M.	SD			
**Demographic variables**	**Age**		13.39	11.04	14.60	10.85	61.00	−1.192	0.139
	**Intelligence**		14.31	11.01	15.63	10.64	85.50	−0.420	0.362
	**Socioeconomic class**		8.80	13.24	7.16	9.3	22.2	−1.12	0.136
**Scales**	**Empathy**		13.21	11.8	14.67	12.2	70.00	−1.13	0.137
	**Cyberbullying**	**Victimization**	24.39	5.59	25.03	5.36	85.50	−1.03	0.938
		**Perpetration**	25.16	5.19	25.98	5.08	84.35	−1.01	0.876

**Table 2:** Counselling methods used in the program.

Definition	Counseling Methods Used	N
A collection of opinions is exchanged between the researcher and the participants to recognize the positive and negative aspects among each other and to train them to freely express their feelings.	Discussion and dialogue	1
This is considered one of the methods that assesses the ability of the participants in the counseling sessions.	Feedback	2
The incidents that participants had in daily life through narration.	The story	3
When one participant acts the role of another to clarify the image and meaning.	Role-play	4
Interactions and opinions among participants of the counseling group.	Exercises	5
Adding fun and cheerfulness to move from anguish to fun and pleasure.	Fun and humor	6
Fictional styles accompanied by images of achievements, superiority, developing self-esteem and improving the quality of life for a group of difficulties in reading.	Imaginary modeling	7
Discussing individuals who succeeded in achieving their goals by their strong ability.	Live to model	8
Where the mentor encourages positive behavior done by participants	Positive reinforcement	9
A discussion that solves any problem through collecting all the spontaneous ideas of the participants.	Brainstorming	10
Engaging the group participants to do homework.	Homework	11

**Table 3:** Mean scores in the experimental group on empathy and cyberbullying before the intervention and immediately after the intervention (N = 217).

Variables	Measurement		Pre-Testing		Post-Testing		*t*-Test	*p*-Value
			M.	SD	M.	SD		
**Empathy**	**Control**		13.21	11.8	16.41	11.22	4.18	0.0021 **
	**Intervention**		14.67	12.2	32.21	5.33		
**Cyberbullying**	**Victimization**	**Control**	24.39	5.59	22.46	3.98	10.99	0.0001 **
		**Intervention**	25.03	5.36	15.62	2.25		
	**Perpetration**	**Control**	25.16	5.19	22.48	4.10	9.982	0.0001 **
		**Intervention**	25.98	5.08	16.00	2.34		

** Significant at the *p* < 0.01 level (2-tailed).

**Table 4:** Mean scores of adolescents in the experimental group on the post- and follow-up measurement on the TEQ and BCS-A (N = 98).

Tests	Measurement		M.	SD	*t*-Test	*p*-Value
**Empathy**	Post-test		32.21	5.33	0.36	0.359
	follow-up		32.18	5.61		
**Cyberbullying**	Victimization	Post-test	15.62	2.25	0.37	0.356
		follow-up	15.46	2.03		
	Perpetration	Post-test	16.00	2.34	0.39	0.348
		follow-up	16.30	2.41		

**Table 5:** Mann-Whitney U test of males and females in the experimental group in the post-measurement of the TEQ and BCS-A (N = 98).

Tests			N.	Mean	SD	Mean Rank	Sum of Ranks	U	Z	*p*-Value
**Empathy**		Males	45	28.56	0.813	23.71	1067.00	32.00	8.44	0.000 **
		Females	53	31.23	0.750	71.40	3784.00			
**Cyberbullying**	**Victimization**	Males	45	13.80	0.694	30.32	1364.50	329.500	6.442	0.000 **
		Females	53	15.02	0.746	65.78	3486.50			
	**Perpetration**	Males	45	14.84	0.95	67.10	3019.50	400.500	5.922	0.000 **
		Females	53	13.42	1.12	34.56	1831.50			

** Significant at the *p* < 0.01 level (2-tailed).

## Data Availability

All data used in this study are available from the corresponding author upon reasonable request.
